# A Novel Parameter Initialization Technique Using RBM-NN for Human Action Recognition

**DOI:** 10.1155/2020/8852404

**Published:** 2020-09-10

**Authors:** Deepika Roselind Johnson, V. Rhymend Uthariaraj

**Affiliations:** ^1^DCSE, CEG–Anna University, Guindy, Chennai, India; ^2^RCC, CEG–Anna University, Guindy, Chennai, India

## Abstract

Human action recognition is a trending topic in the field of computer vision and its allied fields. The goal of human action recognition is to identify any human action that takes place in an image or a video dataset. For instance, the actions include walking, running, jumping, throwing, and much more. Existing human action recognition techniques have their own set of limitations when it concerns model accuracy and flexibility. To overcome these limitations, deep learning technologies were implemented. In the deep learning approach, a model learns by itself to improve its recognition accuracy and avoids problems such as gradient eruption, overfitting, and underfitting. In this paper, we propose a novel parameter initialization technique using the Maxout activation function. Firstly, human action is detected and tracked from the video dataset to learn the spatial-temporal features. Secondly, the extracted feature descriptors are trained using the RBM-NN. Thirdly, the local features are encoded into global features using an integrated forward and backward propagation process via RBM-NN. Finally, an SVM classifier recognizes the human actions in the video dataset. The experimental analysis performed on various benchmark datasets showed an improved recognition rate when compared to other state-of-the-art learning models.

## 1. Introduction

Human action recognition [1] is used for a variety of applications such as video surveillance [2], retrieval [3, 4], and detection [5–7]. The action recognition is performed by computational algorithms [8–10] that understand and detect human actions. These computational algorithms generate a label after detecting a human action. Action recognition involves extracting and learning human actions [11–13]. It can be performed by using three techniques—traditional design features, deep learning, and hybrid extraction [14]. Among these techniques, the hybrid extraction technique [15] has gained prominence in recent years. It involves using both traditional and deep learning techniques for recognition.

In traditional methods [16–20], artificial actions such as spatial convolutions [21, 22], temporal convolutions, and fusion techniques are used for extraction and recognition. Though they provide a good recognition rate, there have been no recent advances. Action recognition is comprised of two components: representation [23–27] and classification [25]. The human actions in a video sequence are generated as a space-time feature in 3D representation [28, 29]. They are comprised of both spatial and dynamic information; the spatial information includes human pose, and dynamic information includes motion. The movement is captured through anchors or bounding boxes to detect the subject from cluttered backgrounds. To capture the spatial-temporal features in human actions, various methods use Poisson distribution to extract the shape features [30, 31]. For action representation and classification, the spatial-temporal information is taken as input. The spatial-temporal saliency is computed from the moving parts and the local orientation is determined. These local representations are converted into global features by computing the weighted average of each point inside the bounding box and analyzing the different geometrical properties [32, 33].

Initially, the spatial-temporal points were extracted using Laptev's [23] and Harris corner detector [24] in the spatial-temporal domain. Gaussian kernel [34] is applied to the video sequence to obtain a response function for the spatial-temporal dimensions. Other prominent methods such as 2D Gaussian smoothing [35] were applied for obtaining the spatial features, and 1D Gabor filter is applied for obtaining the temporal features along with other information such as raw pixels, gradient, and flow features. Principal component analysis [36–38] is applied to the vector features for dimensionality reduction. The detection algorithms such as 3D SFIT [39], HOG3D [7, 40], HOG [41], and HOF [41] are used for describing the trajectories [42–44].

The spatial-temporal point of interest [45] captures only short-term distance. However, to describe the change in motion, it is necessary to track the points continuously. The trajectories along with the interest points are detected and tracked using Haris3D [24] with the KLT tracker [46]. Using this method [47], the trajectories are mapped with corresponding SIFT points over consecutive frames. Using the HOG, HOF, and MBH [48] features, the intertrajectories and intratrajectories are described. After the action is represented, action classifiers [30, 31, 45, 49–51] are applied to the training samples to determine the class boundaries. The human actions are classified into two types: direct classification and sequential method. The direct classification involves the extraction of a feature vector and recognition of actions from classifiers using SVM [36] and K-NN method [52, 53]. In the sequential method, the temporal features such as appearance and pose are obtained from the hidden Markov model [54–56], conditional random fields [57–60], and structured support vector machine [61–64]. Furthermore, representative key poses are learned for efficient representation of human actions [33, 34, 65–72] to build a compact pose sequence.

Deep learning techniques [73] such as 2D ConvNets [21, 74] and 3D ConvNets [26] perform feature learning via convolution operator and temporal modeling [75]. The initialization of a deep neural network [72] is crucial for training the model. To ensure that the state of the hidden layers follow a uniform distribution, a model parameter [76–78] is initialized. If the model parameter [79, 80] is not properly initialized, it leads to gradient explosion. The most commonly used technique is the Xavier initialization method [81] modeled based on the sigmoid activation function. Many models use ReLU activation function [82], RBMs [83, 84], and other methods [85] for learning.

In this paper, we propose a novel parameter initialization technique using the Maxout activation function (MAF) via restricted Boltzmann machine-neural network (RBM-NN).

The spatial and temporal features required for human action recognition are obtained from the video sequence via a feature learning process. The extracted spatial and temporal features are trained using RBM-NN. The RBM-NN converts the local features into global features using an integrated forward and backward propagation process. An SVM classifier is used for recognizing the human actions in the video sequence.

Section 2 describes the process of tracking human action from video sequences, extraction of shape features, and construction of an RBM-NN.  Section 3 describes parameter initialization using an activation function, forward propagation, backward propagation, and action recognition using an SVM classifier.  Section 4 consists of data preprocessing and model training for analyzing the effectiveness of the parameter initialization technique.  Section 5 discusses the experimentation setup, result analysis performed on various benchmark datasets, influence of the learning parameter on model accuracy, and the loss function. Finally, Section 6 consists of concluding remarks followed by references.

## 2. Methodology

**Figure 1:**
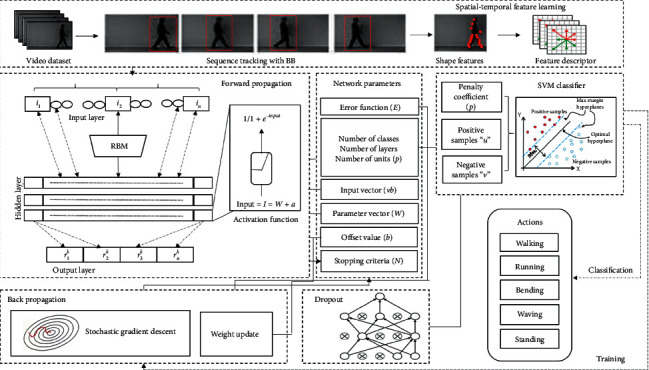
Proposed methodology for human action recognition.

The spatial-temporal features [86, 87] for human action recognition are performed via a feature learning process [59, 62], as shown in Figure 1. The first step involves using detection and sequence tracking algorithm [88] to identify human action features. Secondly, the action tracking sequence is segregated into blocks to extract the shape features using the neural network layers implemented by RBM [83, 89]. The model is implemented by dividing the network layers and feeding the output of the first layer as input to the second layer to learn the spatial-temporal features. The second hidden layer is used for dimensionality reduction of the output from the first layer and to reduce computational efficiency.

### 2.1. Human Action Tracking from Video Sequence

The action changes in the human body are detected from video frames by posture and action changes. Target detection and tracking such as pedestrian detection algorithm [90, 91] are used to automatically detect and track the action sequences. A bounding box tracks the subject of interest and is optimized based on pose normalization. From the video dataset, the length of the tracking sequence is set to a fixed length *L*. If the length of the initial tracking sequence is greater than *L*, the redundant frames are discarded. If the length of the initial tracking sequence is lesser than *L*, the tracking sequence is extended by the zero-padding method and is set to *L* frames. The human actions from the tracking sequence are denoted by *a*_*i*_, and other actions are denoted as *o*_*i*_.

### 2.2. Extracting Shape Features

Every tracking sequence is divided into video blocks, and the initialization parameters are specified as *vb*_*w*_ ×  *vb*_*h*_. The segregated blocks are denoted as *V*_*k*_, *k* ∈ *K*,  *where*  *K*={1,2,…, *V*_*k*_^*vb*_*w*_×*vb*_*h*_^} corresponds to the spatial position of the block. In the proposed method, a deep neural network is used for extracting the spatial-temporal features from low-level features. The first step involves segregating blocks into individual frames *B*_*k*_, *k* ∈ *K*, *B*_*n*_^*k*^,  *where* *n*=1,2,…, *L* into grid cells *C*_*w*_ ×  *C*_*h*_. Each grid cell is computed in *C*_*d*_ directions in the histogram of oriented gradients (HOGs) and represents the shape characteristics. The shape dimensions of each image frame are denoted as *S*_*w*_ ×  *S*_*h*_ ×  *S*_*w*_. The feature vector is represented as (*s*_*m*1_^*k*^, *s*_*m*2_^*k*^,…, *s*_*nm*_^*k*^),  *where* *m*=*S*_*w*_ × *S*_*h*_ × *S*_*w*_. The initial component of the shape feature of the image frame IF_*m*_^*k*^ is indicated as *s*_*nt*_^*k*^,  *where* *t*=1,2,…, *n*. The shape features from each block are extracted and divided into a long vector. These individual feature vectors represent the shape features.

During action recognition, the pose of the person is estimated and the shape features are extracted from the tracking sequence. The extracted shape features, i.e., pose in individual frames are normalized. The frame from a tracking sequence is represented as *V*_*k*_^1^, *V*_*k*_^2^,…, *V*_*k*_^*vb*_*w*_×*vb*_*h*_^,  *where* *k*=1,2,…, *n*. The normalized shape vectors for every frame in the tracking sequence are given as

(1) $$\begin{array}{c} I_{nt}^{k}=\frac{s_{nt}^{k}}{{\left({\sum_{k=1}^{vb_{w}\times vb_{h}}\sum_{l=1}^{s}\left|s_{nl}^{k}\right|}^{2}\right)}^{\left(1/2\right)}}, \end{array}$$

where 1 ≤ *t* ≤ *s*, *I*_*nt*_^*k*^ is the normalized shape feature vector and the component *s*_*nt*_^*k*^ is the shape factor vector that corresponds to the normalized value. The shape feature for every individual frame in the block is denoted as *B*_*n*_^*k*^=(*l*_*n*1_^*k*^, *l*_*n*2_^*k*^,…, *l*_*nm*_^*k*^),  *where* *k*  ∈ *K*, 1 ≤ *n* ≤ *L*. The shape features from the video block are represented as *B*_1_^*k*^, *B*_2_^*k*^,…, *B*_*L*_^*k*^. The dimensional features are represented as *L* × *S*_*w*_ × *S*_*h*_ × *S*_*w*_*·l*_*nt*_^*k*^ ∈ [0,1]. The eigenvectors of the shape features are denoted as *B*_1_^*k*^, *B*_2_^*k*^,…, *B*_*L*_^*k*^ and is provided as input to train the RBM-NN.

### 2.3. Constructing an RBM-Neural Network

**Figure 2:**
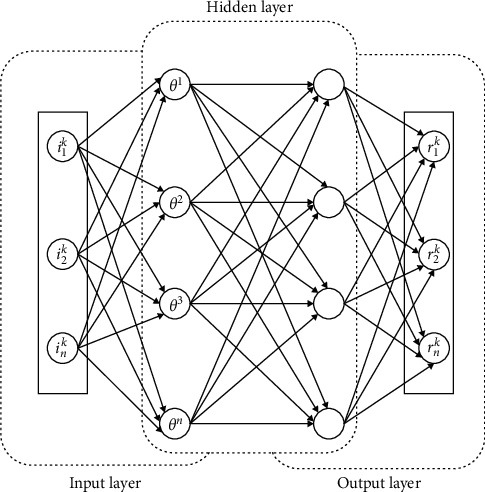
Multiple restricted Boltzmann machines.

Restricted Boltzmann machine [54, 63] is comprised of a network architecture that consists of two neuron layers: the input layer and the hidden layer. The nodes present in the input layer and hidden layers are connected, but they are connected with a particular layer. RBMs are capable of self-learning through discrete distribution via the hidden neutrons. The input layer consists of multiple RBMs, as shown in Figure 2, to describe the distribution of action characteristics. For each type of action category, the training samples are fed to the RBMs with spatial features.

The output layers from each RBM comprise of *N* neurons, and the value of *N* has a direct influence on the distribution of every action learned. The proposed method analyses influence that the value *N* has on the experimental results. For every RBM present in the neuron network layer, the limits are set as *k*=1,…, *vb*_*w*_ × *vb*_*h*_. It is used for training the various shape features from the blocks along with their corresponding spatial position ′*k*′ as input. The input video block has the following shape feature *I*^*k*^=(*i*_11_^*k*^, *i*_12_^*k*^,…,*i*_*Ln*_^*k*^)^*L*^, and the corresponding output is represented as *R*_*k*_=*R*_*k*_=(*r*_1_^*k*^, *r*_2_^*k*^,…,*r*_*N*_^*k*^)^*L*^. The restrictions in the RBM-NN, its state, and energy of the neurons {*I*_*k*_, } is defined as

(2) $$\begin{array}{c} E\left(I^{k},R^{k};\theta^{k}\right)=-{\left(I^{k}\right)}^{L}P^{k}R^{k}-{\left(b^{k}\right)}^{L}I^{k}-{\left(a^{k}\right)}^{L}R^{k}\\ =-\sum_{n=1}^{L\times m}\sum_{t=1}^{T}P_{nt}^{k}I_{n}^{k}R_{t}^{k}-\sum_{n=1}^{L\times m}b_{n}^{k}I_{n}^{k}-\sum_{t=1}^{T}a_{t}^{k}R_{t}^{k}, \end{array}$$

where *θ*^*k*^= {*P*^*k*^} in which *θ*^*k*^ is the RBM parameter and *P*^*k*^ represents the symmetric correlation between the input and output neurons. Also, *a*_*k*_ and *b*_*k*_ indicate the deviation among the column vectors generated in the input and the output layer. The set of model parameters used in RBM is learned using the contrastive divergence (CD) algorithm [92]. The CD algorithm is effective for training undirected graphical models (RBMs) and estimates the energy gradient given a set of model parameters along with the training data. The CD provides the gradient estimates and enables the model to keep balanced and avoids the issue of gradient explosion and overfitting. The distribution between the input and output neurons for a single RBM is given as

(3) $$\begin{array}{c} G\left(I^{k},R^{k};\theta^{k}\right)=\frac{1}{Z\left(\theta^{k}\right)}\exp\left(-E\left(I^{k},R^{k};\theta^{k}\right)\right). \end{array}$$

(4) $$\begin{array}{c} Z\left(\theta^{k}\right)=\sum_{I^{k}}\sum_{R^{k}}\exp\left(-E\left(I^{k},R^{k};\theta^{k}\right)\right), \end{array}$$

where *θ*^*k*^ is the partition function and the conditional probability distribution is derived from equation (3):

(5) $$\begin{array}{c} g\left(R_{t}^{k}=1\left|I^{k}\right.\right)=h\left(\sum_{n}W_{nt}^{k}\;I_{n}^{k}+a_{t}^{k}\right),\\ g\left(I_{N}^{k}=1\left|R^{k}\right.\right)=h\left(\sum_{t}W_{nt}^{k}R_{n}^{k}+b_{t}^{k}\right). \end{array}$$

The proposed method trains the RBM in the first layer of the neural network architecture. The network parameter set of the multiple RBM neural network layers for every action is denoted as *θ*=*θ*^1^, *θ*^2^,…, *θ*^*n*^.

The proposed work is used for training the two-layer neural network for every action category. The second layer of the neural network is also an individual RBM and solely used for dimensionality reduction of the output obtained from the first layer. The parameter of the network layer is denoted as (*W*, *a*). For every action category, the input from an action sequence will provide the feature vectors as output.

The output of the trained two-layered neural network is modeled based on spatial-temporal shape feature learning from the block. The spatial-temporal individualities are represented as *R*=(*r*_1_, *r*_2_,…, *r*_*A*_),  *where* *A* is the set generated based on experience and is denoted as *A*=16 × *vb*_*w*_ × *vb*_*h*_ × *N*.

## 3. Parameter Initialization Using Activation Function

### 3.1. Importance of Effective Parameter Initialization

To build an efficient model for human action recognition, an RBM-NN architecture is defined in the proposed work and it is trained to learn the parameters. The RBM-NN architecture is trained using the following steps: parameter initialization, optimization algorithm, forward propagation, cost function computation, gradient cost computation using back propagation, and parameter updation.

When testing data are provided, the network uses the trained model to predict the class. For a network to perform efficiently, it is crucial to initialize the right parameter to avoid the problem of gradient explosion and vanishing.

**Case 1 .**

If the initialized parameter is large, it leads to a gradient explosion:

(6) $$\begin{array}{c} \text{initialized }\mathrm{weight}\;\gg \;\mathrm{identity}\text{ matrix}. \end{array}$$

**Case 2 .**

If the initialized parameter is small, it leads to vanishing gradients:

(7) $$\begin{array}{c} \text{initialized }\mathrm{weight}\;\ll \;\mathrm{identity}\text{ matrix}. \end{array}$$

To prevent the problem specified above, a set of rules have to be adhered to while initializing the network parameter. Initially, the mean value of the activation function must always be zero. Finally, the variance of the activation function must remain uniform throughout the network layers. If the rules are not followed, it gives rise to a locally optimal solution which renders the model untrainable and improper feature extraction.

The model parameter is initialized based on two categories: parameter initialization by pretraining a model and parameter optimization by training the neural network. In the first method, a model is trained using the unsupervised model, and an AutoEncoder [93] is used to build a layer-by-layer unsupervised objective function. The layer-by-layer training is performed on equal depth neural networks to obtain the feature representations from the input. Pretraining a model involves computational overhead, and the training efficiency is affected. The second method involves initializing the parameter and its optimization using neural networks. The parameter can be initialized using a nonlinear activation function and backpropagation.

### 3.2. Parameter Initialization Using Maxout Activation Function

In this paper, the parameter initialization technique is modeled using a Maxout layer. The layer consists of an activation function which takes the maximum of the inputs. When compared to other activation functions, Maxout activation function [94] performs well due to the dropout technique. Dropout is a model averaging technique where a random subnetwork is trained for every iteration and the weights are averaged at the end. An approximation has to be used as these weights cannot be averaged explicitly. The inputs to the Maxout layer are not dropped using the corresponding activation function. The input with the maximum value for the data point is not affected as the dropout occurs in the linear part. Thus, it leads to efficient model averaging as the averaging approximation is for linear networks.

In the proposed work, it is assumed that the state of the neuron node follows a uniform distribution required for a Maxout activation function. It is an activation function that is capable of training itself in our model. It performs a piecewise linear approximation on ReLU, absolute function, and quadratic function to a random convex function. It considers the maximum value from a set of linear values that are determined beforehand. The Maxout implements ReLU and absolute function using two linear functions and the quadratic function using four linear functions. It can approximate any function using multiple linear functions and is known as piece-wise linear approximation.

The Maxout unit is implemented using the following function:

(8) $$\begin{array}{c} f\left(x\right)=\max\left(w_{1}x+b_{1},w_{2}x+b_{2},\ldots ,w_{n}x+b_{n}\right), \end{array}$$

where *n* is the number of linear combinations. If *w*_1_ is set to one, all the other values take the value zero such that the proposed activation function becomes equivalent to the traditional activation functions.

As mentioned earlier, any continuous piece-wise linear approximation can be expressed as a difference between two convex functions:

(9) $$\begin{array}{c} g\left(x\right)=f_{1}\left(x\right)-f_{2}\left(x\right), \end{array}$$

where *f*_1_(*x*) and *f*_2_(*x*) are the convex functions and *g*(*x*) is a continuous piece-wise linear approximation function. From equation (9), it can be deduced that a Maxout layer comprising two Maxout units can be used to approximate any continuous function randomly.

Also, both ReLU and leaky ReLU are considered to be special cases of a Maxout unit and enjoy all the benefits of a ReLU unit. It implements linearity of operations with no saturation and avoids the issue of dying ReLU. A Maxout can be formed with more units, but this will increase the capacity of the network and requires more training. Thus, Maxout units are considered as universal approximators.

The MAF is modeled based on theoretical derivation for parameter initialization of the model. Both forward propagation and backward propagation process in the network are analyzed to ensure that every neuron follows a uniform distribution.

### 3.3. Forward Propagation Process

To perform forward propagation, the following assumptions are made: (1) the input vector *vb* and the parameter vector *W* must be independent; (2) the input vector *vb* and the parameter vector *W* must follow the same distribution; (3) the initial distribution of the parameter vector *W* must be symmetrical about the zero-point; and (4) the offset value *b* of each layer must always be zero.

The response of the hidden convolution layer in the RBM-NN is given as

(10) $$\begin{array}{c} z_{t}=x_{t}^{L}\;W_{t}+b_{t}, \end{array}$$

where *t* denotes the *n*^th^ hidden layer of the RBM-NN, among which *x*_*t*_ ∈ *A*_*p*_,  *x*^*t*^ is the original input vector, and the mean value is set to zero after processing.

(11) $$\begin{array}{c} p=u^{2}i, \end{array}$$

where *p* is the number of input nodes connected to one neuron node, *u* is the size of the convolution kernel, and ′*i*′ is the number of input channels to the model. The output of every neuron node is passed through the MAF provided as follows:

(12) $$\begin{array}{c} f\left(x\right)=\max\left(w_{1}x+b_{1},w_{2}x+b_{2},\ldots ,w_{n}x+b_{n}\right), \end{array}$$

where *n* is the number of linear combinations. If *w*_1_ is set to one, all the other values take the value zero such that the proposed activation function becomes equivalent to the traditional activation functions. The problem of local linearity in the proposed activation function eliminates the issue of gradient explosion, but there is an increase in computational overhead during the training process.

The variance of the initialization parameter can be obtained as follows:

(13) $$\begin{array}{c} \text{Var}\left[z_{t}\right]=p_{t}\text{Var}\left[W_{t}x_{t}\right]. \end{array}$$

The weight *W*_*t*_ and hidden layers have to adhere to Gaussian distribution with a mean value of zero as per assumptions 2 and 3. The initial state and the parameter vectors are assumed to be independent of each other as per assumption 1. Thus, the variance in the initialization parameter is provided:

(14) $$\begin{array}{c} \text{Var}\left[z_{t}\right]=p_{t}\text{Var}\left[W_{t}\right]E\left[x_{t}^{2}\right], \end{array}$$

where *E*[*x*_*t*_^2^] is the exception function. The proposed activation function can be simplified by considering two linear functions given as follows:

(15) $$\begin{array}{c} x_{t}=r_{t-1}\left(x_{t-1}\right)=\max\left(z_{t-1,1},z_{t-1,2}\right). \end{array}$$

Based on assumption 4, the offset value *b*_*t*−1_ is always set to zero and the mean weights *W*_*t*_ are also set to zero. The values *z*_*t*−1,1_, *z*_*t*−1,2_ are assumed to be symmetrical at the mean point and follow the same distribution.

The expectation function *E*[*x*_*t*_^2^] and the variance Var[*z*_*t*−1_] are defined as follows:

(16) $$\begin{array}{c} x_{t}=\frac{z_{t-1,1}+z_{t-1,2}+\left|z_{t-1,1}-z_{t-1,2}\right|}{2}. \end{array}$$

The expectation *E*[*x*_*t*_^2^] value is obtained by substituting equation (15):

(17) $$\begin{array}{c} E\left[x_{t}^{2}\right]=\frac{1}{2}\left(\text{Var}\left[z_{t-1,1}\right]+\text{Var}\left[z_{t-1,2}\right]\right). \end{array}$$

As per assumption 2, the values *z*_*t*−1,1_ and *z*_*t*−1,2_ follow the uniform distribution and the new variance is obtained as follows:

(18) $$\begin{array}{c} \text{Var}\left[z_{t-1}\right]=\text{Var}\left[z_{t-1,1}\right]=\text{Var}\left[z_{t-1,2}\right]. \end{array}$$

Substituting the variance value obtained from equation (17) into equation (16), we get

(19) $$\begin{array}{c} E\left[x_{t}^{2}\right]=\text{Var}\left[z_{t-1}\right]. \end{array}$$

The relationship between the variances is obtained by substituting equation (17) into equation (13) as follows:

(20) $$\begin{array}{c} \text{Var}\left[z_{t}\right]=p_{t}\text{Var}\left[W_{t}\right]\text{Var}\left[z_{t-1}\right]. \end{array}$$

The difference in variance between the first hidden layer and the last hidden layer is obtained as follows:

(21) $$\begin{array}{c} \text{Var}\left[z_{T}\right]=\left(\prod_{t=2}^{T}p_{t}\text{Var}\left[W_{t}\right]\right)\text{Var}\left[z_{t}\right]. \end{array}$$

The initialization parameter for a neural network model must follow the necessary condition:

(22) $$\begin{array}{c} p_{t}\text{Var}\left[W_{t}\right]=1,\;\forall t. \end{array}$$

When *t* is set to 1, equation (21) is satisfied without the interference on the input vector by the activation function. Based on the theoretical assumption, each node in the hidden layer behaves similarly to a neural network. Also, the model parameter initialization for every node in the hidden layer satisfies the Gaussian distribution.

### 3.4. Backpropagation Process

In backpropagation, the following assumptions are made similar to forward propagation: (1) the gradient Δ*r*_*t*_ and the parameter vector *W* must be independent of each other; (2) the gradient Δ*r*_*t*_ and the parameter vector *W* must follow the same distribution; and (3) the gradient Δ*r*_*t*_ and the parameter vector *W* must have zero symmetry for *E*[Δ*x*_*t*_]=0.

The concentration of gradients obtained by the convolution parameter is shown as follows:

(23) $$\begin{array}{c} \Delta x_{t}=W_{t}\Delta^{\Delta }r_{t}, \end{array}$$

where Δ*x*_*t*_ and Δ*r*_*t*_ are the gradients that represent the loss functions. The value of the activation function is obtained when *a*=0:

(24) $$\begin{array}{c} \Delta z_{t},n=f^{\prime }\left(z_{t},n\right)\Delta \;x_{t+1},\;n\in \left\{1,2\right\}. \end{array}$$

If *f*′(*z*_*t*_, *n*)=1 and *f*′(*z*_*t*_, *n*)=0, each has half probability of occurrence. Moreover, *f*′(*z*_*t*_, *n*)=1 and Δ*x*_*t*+1_ are independent of each other based on assumption 1.

The initial condition *n* ∈ {1,2} is provided:

(25) $$\begin{array}{c} E\left[\Delta r_{t}\right]=E\left[\Delta x_{t},n\right],\\ E\left[{\left(\Delta r_{t}\right)}^{2}\right]=\text{Var}\left[\Delta r_{t}\right]=\frac{1}{2}\text{Var}\left[\Delta x_{t+1}\right]. \end{array}$$

The variance function for the gradient is obtained as follows:

(26) $$\begin{array}{c} \text{Var}\left[\Delta x_{t}\right]=\frac{1}{2}r\wedge_{t}\text{Var}\left[W_{t}\right]\text{Var}\left[\Delta x_{t+1}\right]. \end{array}$$

The relationship between Var[Δ*x*_2_] and Var[Δ*x*_*T*+1_] can be defined as follows:

(27) $$\begin{array}{c} \text{Var}\left[\Delta x_{2}\right]=\text{Var}\left[\Delta x_{T+1}\right]\left(\prod_{t=2}^{T}\frac{1}{2}r\wedge_{t}\text{Var}\left[W_{t}\right]\right). \end{array}$$

For the gradient to move smoothly, the following initial condition has to be satisfied:

(28) $$\begin{array}{c} \frac{1}{2}r\wedge_{t}\text{Var}\left[W_{t}\right]=1,\;\forall t\;\in \left[2,T\right]. \end{array}$$

The parameter for neural network model *W* also follows the same distribution based on assumption 2:

(29) $$\begin{array}{c} W_{t}∼N\left(0,\frac{2}{r\wedge_{t}}\right). \end{array}$$

It is not possible to perform both forward and backward propagation at the same time. Thus, the parameter has to be optimized as follows:

(30) $$\begin{array}{c} \min_{\tau_{t}}{\left(\tau_{t}-r_{t}\right)}^{2}+{\left(\tau_{t}-\;\frac{1}{2}r\wedge_{t}\right)}^{2}. \end{array}$$

The optimized solution for the proposed initialization parameter for RBM-NN based on uniform distribution is obtained:

(31) $$\begin{array}{c} W_{t}∼N\left(0,\frac{4}{2r_{t}+r\wedge_{t}}\right). \end{array}$$

### 3.5. SVM Classifier for Action Recognition

An SVM classifier is built for each action category. The training of the RBM-NN is categorized into two samples: positive samples and negative samples. The samples which correspond to action categories *a*_*i*_ are classified as positive samples ′*u*′ and other actions *o*_*i*_ as negative samples ′*v*′. The parameter vector *W* and the other variables are optimized. If there is an imbalance in the positive and negative samples, the classification accuracy in the training phase is affected. To overcome the issue of accuracy, a penalty coefficient parameter ′*P*′ is introduced. If the training set has less positive samples, a higher penalty coefficient *P* is enforced and the negative samples are introduced to a lesser penalty coefficient  *P*.

The SVM objective function for our proposed method is defined as follows:

(32) $$\begin{array}{c} \begin{array}{cc} \min_{\omega ,\varepsilon } & \frac{1}{2}{\left\|\omega \right\|}^{2}+P+\sum_{i=1}^{u}\varepsilon_{i}+P-\sum_{j=u+1}^{u+v}\varepsilon_{j}\\ \text{s}.\text{t}.\; & y_{i}\left[\left(\omega^{L}R_{i}\right)+b\right]\geq 1-\varepsilon_{i},\;i=1,2,\ldots ,u+v,\\ & \varepsilon \;\geq 0, \end{array} \end{array}$$

where *i*=1,2,…, *u*+*v*, *R*_*i*_ is the spatial-temporal feature of the *i*^th^ action sample and (*R*_*i*_, *y*_*i*_) is the input of the SVM classifier. Also, *u*+*v* is the total number of training samples used for training the SVM classifier. The SVM classifier is trained for each action category and represented as an action model (*θ*, *W*, *a*, *b*) comprising two-layer RBM-NN for human action recognition.

## 4. Result Analysis and Discussion

The parameter initialization proposed in the paper is verified and analyzed on the MS-COCO [95], ImageNet [96], and CIFAR-100 [97] datasets respectively. The RBM-NN comprises four convolution layers for analysis along with the loss function. The loss function considered in the model is the logistic loss layer obtained after downsampling. To prevent overfitting, the dataset is separated into batches and trained as submodels. The parameter is initialized randomly, and the submodels are trained using the dropout technique by randomly setting the output nodes to zero before updating the training set. The dropout probability for the model validation is set as 50% to determine the classification error rates.

### 4.1. Data Preprocessing

The training data are preprocessed by applying global contrast normalization and zero component analysis whitening [98]. The GCN technique prevents the images from exhibiting various levels of contrast. The mean value is subtracted, and the image is rescaled such that the standard deviation across the pixels is constant. ZCA whitening process ensures that the average covariance between the whitened pixel and the original image is maximal. For instance, it makes the data less redundant by removing the neighboring correlations in adjacent pixels.

### 4.2. Model Training

The models were initially trained using the Xavier initialization method [81] for parameter initialization and the model parameters. The Xavier initialization method is chosen since it keeps the variance uniform across each network layer as per the assumptions followed during the forward propagation process. The initial and model parameters must follow a uniform distribution specified below:

(33) $$\begin{array}{c} W∼U\left[-\frac{\sqrt{6}}{\sqrt{n_{k}+n_{k+1}}},\right]\frac{\sqrt{6}}{\sqrt{n_{k}+n_{k+1}}}, \end{array}$$

where *n*_*k*_ is the number of input nodes and *n*_*k*+1_ are the number of output nodes. The datasets MS-COCO [95], ImageNet [96], and CIFAR-100 [97] were considered as input for the proposed parameter initialization method and also compared with parameter initialized via the Xavier model. The proposed parameter initialization method showed similar results in the classification accuracy of the activation function. The improvement in classification accuracy has been attributed to the fact that nodes and states of the various hidden layers follow the same distribution pattern and avoids the problem of gradient explosion.

The dataset ImageNet comprises a 1000-class image problem and required 120 epochs. The MS-COCO comprises 80 classes and required 64 epochs for training. The CIFAR-100 dataset is comprised of 100 classes and required 200 epochs for training. The model required more layers for analysis along with the introduction of convolution kernels. The deep neural network model was able to perform iteration for 500,000 times with a learning rate set to 0.1. However, it was found that the learning rate decreased with an increase in the number of iterations. The comparison of the test error rates between the proposed initialization method and the Xavier initialization method is provided in Table 1. The analysis shows that the error rates obtained from the proposed method showed better results for both small (MS-COCO) and large datasets (ImageNet and CIFAR-100).

The model parameters along with the slack variables are initialized and optimized by the objective function used by the SVM classifier. During the training process, it was noticed that there was an imbalance between the positive and negative samples.

For instance, there were fewer positive samples in the training set when compared to the negative samples. Thus, a higher penalty coefficient ′*P*′ was introduced to the positive samples to balance the training samples.

## 5. Experimentation Setup and Analysis

The human action recognition using the proposed method is performed using the datasets specified in Table 2 along with their classes, modalities, and environment type. These benchmark datasets are comprised of actions performed in both simple and cluttered background scenes. The datasets are divided into training and testing sets. This discriminative action is used for segmentation to reduce the background correlation between the training and the testing set. The model is trained using small samples, and the data expansion method [108] is used increasing the number of video samples present in the training set.

Initially, the actions are detected from the video blocks to extract the spatial-temporal features. The features are fed to the RBMs for training along with suitable model parameters via forward and backward propagation process. The output from the RBMs is fed to the SVM classifier for human action recognition. During the experiment analysis performed on the dataset, the influence of the *N* parameter is analyzed along with the penalty coefficient  *P*. The effect of the number of output neurons for each RBM is obtained by adjusting the value of the parameter  *N*. The number *N* of the output neurons is influenced by the average recognition rate of the action sequence. The value of *N* determines the number of spatial-temporal features based on RBM-NN.

The SVM classifier is used for action recognition of multiple types of actions. The SVM classifier model calculates the shape features of the video blocks for each action category. After the classification values are compared, the largest classification value is set as an action label for the test video sequence. The actions from the tracking sequence are detected from the action video.

The proposed algorithm operates on the image sequences with varied focus points, deep learning is used for learning all the features, and SVM classification is performed. The proposed action recognition feature is more specific than other methods. Finally, the model is compared with other state-of-the-art techniques to compare the classification accuracy rate of the model.

### 5.1. Weizmann Dataset

**Figure 3:**
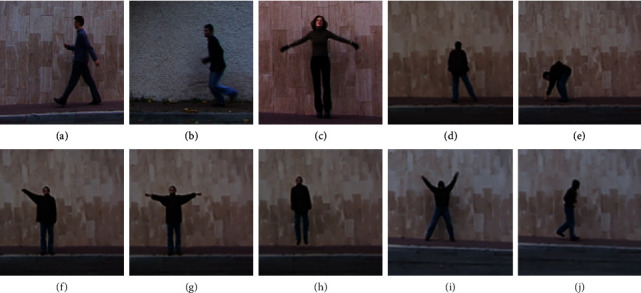
A Weizmann dataset with the actions considered for the proposed approach: (a) walking; (b) running; (c) jumping; (d) galloping; (e) bending; (f) one-hand waving; (g) two-hand waving; (h) jumping in place; (i) jumping jacks; (j) skipping.

The Weizmann dataset [99] is made available by the Weizmann Institute of Science and consists of two datasets. The event-based analysis dataset consists of long sequences of around 6000 frames comprising various people. The actions are divided into four categories: running in place, walking, running, and waving. The ground truth dataset is action annotated for every frame and can be temporally segmented. The second dataset Weizmann actions as space-time shapes dataset was created for human action recognition systems that are suitable for spatial and temporal volumes. The videos were recorded on a simple background with nine persons performing ten actions. The human actions have been divided into ten categories such as walking, running, jumping, galloping, bending, one-hand waving, two-hands waving, jumping in place, jumping jacks, and skipping, as specified in Figure 3. It is a database of 91 low-resolution video sequences. The dataset comprising 91 video sequences is divided into 60 video samples for the training set and 31 action samples for the testing set.

During experimentation, every action in the tracking sequence was divided into 180 × 144 (25 fps) video blocks. The parameter *N* is set to 300, where *N* represents the number of output neurons of each RBM present in the first neural network layer. The proposed method is compared with the reference method [109]. For determining the SVM classifier, set the penalty coefficient *P* = 10, and other slack variables are determined by the objective function. The neural network parameters are obtained by adaptive matching with the processed image data. The proposed work correctly identifies the rotation action of the Weizmann actions as space-time shapes dataset such as walking, running, jumping, bending, waving, and skipping.

**Figure 4:**
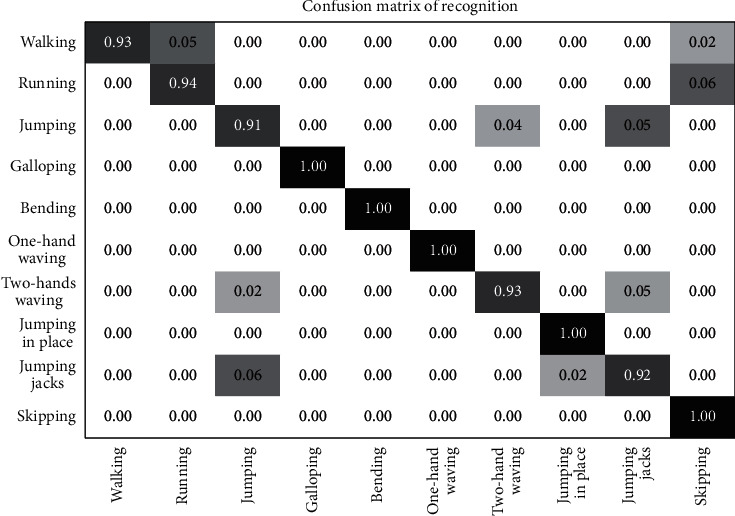
Confusion matrix for the Weizmann dataset.

The proposed method is compared with the reference model [110] proposed by Haiam et al. They proposed a trajectory-based approach for human action recognition to obtain the temporal discriminative features. The trajectories are extracted by detecting the STIPs and matching them with the SIFT descriptors in the video frames. The trajectory points are represented using the bag of words (BoW) model. Finally, an SVM-based approach is used for action recognition. From the confusion matrix shown in Figure 4, it can be noticed that there are some confusions in some frames for actions such as walking, running, jumping, and skipping. Also, the action two-hand waving is similar to jumping jacks. These confusions influence the classification accuracy of the proposed model.

**Figure 5:**
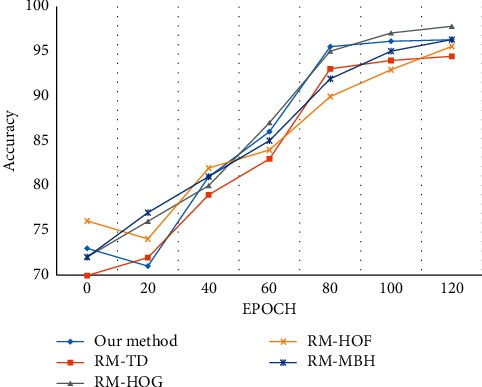
Evaluation of the classification accuracy of the Weizmann dataset.

The proposed approach is evaluated with the classification accuracy obtained by the following descriptors: TD, HOG, HOF, MBH, and the combinations, as shown in Figure 5.  Table 3 shows the average recognition rate for the dataset along with the reference method. It can be noticed that the accuracy rate for the HOG, HOF, and combined features achieved better accuracy when compared to the proposed method due to variations in the codebook sizes and model representation. The vector patches are converted to codewords to produce a codebook comprising similar patches. Moreover, it was observed that the average recognition of the model decreases based on the influence of the number of output neurons.

### 5.2. CAVIAR Dataset

**Figure 6:**
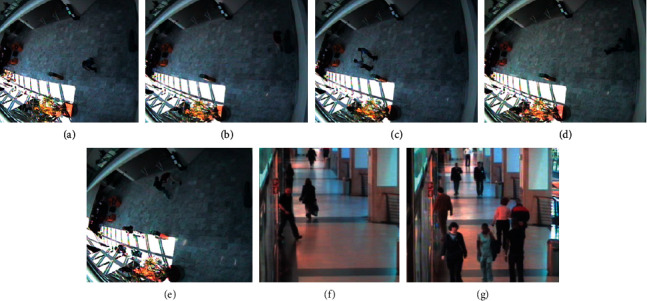
CAVIAR dataset with the sample actions: (a) walking; (b) browsing; (c) meeting; (d) slumping; (e) fighting; (f) exiting; (g) entering.

The context-aware vision using image-based active recognition (CAVIAR) is a video dataset [100]. The dataset consists of seven activities such as walking, slumping, fighting, entering, exiting, browsing, and meeting, as shown in Figure 6. The video sequences were recorded at different locations using a wide-angle camera lens in the INRIA Labs located in France and at a shopping center in Lisbon. The ground truth file is available in the CVML format. The file contains two types of labeling: activity label and scenario label. For every individual, the tracked target comprises 17 sequences and the pixel positions depend on image scaling. The second video sequence displays the frontal view and is synchronized frame by frame. The sequences are 1500 frames longer than the first sequence. The France sequence is categorized as “d1,” and the Lisbon sequence is classified as “d2.”

**Figure 7:**
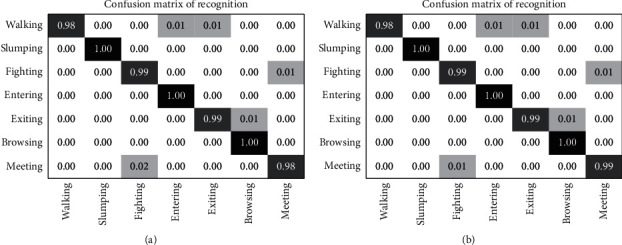
Confusion matrix for the CAVIAR dataset with labels (a) d1 and (b) d2.

The size parameter is set to *N* = 100 and the effectiveness of the recognition method involved classifying two datasets. For the SVM classifier, the penalty coefficient was fixed as  *P* = 10 and other slack variables are fixed by adaptive matching. The training set was categorized into 20 actions for the validation set and 9 actions for the training set. From the confusion matrix shown in Figure 7, it can be seen that some confusions are observed for the actions walking, entering, and exiting. Moreover, similarities were also observed for the actions of fighting and meeting. The other actions in the dataset are classified accurately.

The proposed method was compared with the reference method [112] implemented using the MFS detector and OpenCV classifier.

**Figure 8:**
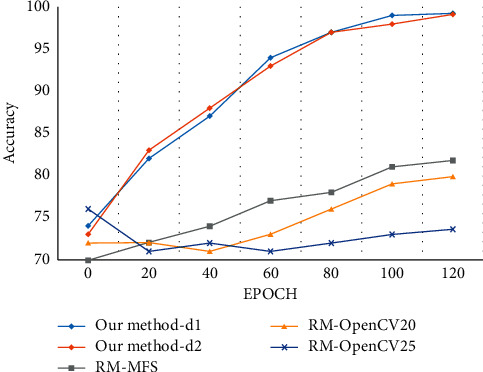
Evaluation of the classification accuracy of the CAVIAR dataset.

The results from Table 4 and Figure 8 show that the recognition rate from our proposed method for both labels “d1” and “d2” is significantly better than the reference method. Negri et al. [112] proposed an approach for pedestrian detection using movement feature space (MFS) to detect the movements and descriptor generation using a cascade of boosted classifiers. The validation of the MFS detector is performed using an SVM classifier. The reference method considered only the frontal view of the dataset resulting in only a few samples used for validation purposes. The less recognition rate achieved by the OpenCV detector 20 (20 stages) and OpenCV detector 25 (25 stages) because both classifiers require more stages for training to reduce the occurrence of false detection.

### 5.3. UCF Sports Action Dataset

**Figure 9:**
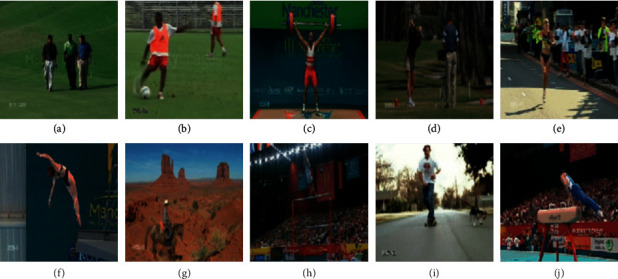
Actions considered for the proposed approach in the UCF sports action dataset: (a) walking; (b) kicking; (c) lifting; (d) golfing; (e) running; (f) diving side; (g) horse riding; (h) swing-side angle; (i) skate boarding; (j) bench swinging.

The UCF sports human dataset [101] is comprised of 150 videos with 10 action categories. The ten categories of actions include walking, kicking, lifting, golfing, running, diving-side, horse-driving, swing-side angle, skateboarding, and bench swinging, as shown in Figure 9. The 150 video samples are divided into 102 samples for the training set and 48 samples for the testing set.

**Figure 10:**
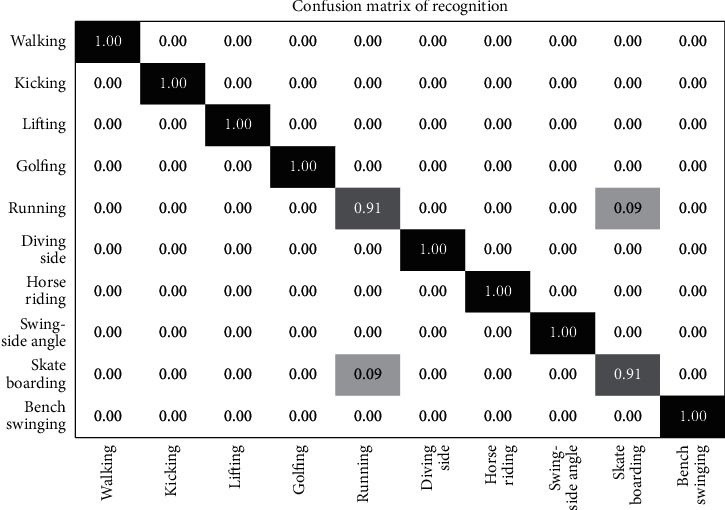
Confusion matrix for the UCF sports action dataset.

The  *N* parameter for each cell is set to 200, and the penalty coefficient is set to  *P* = 10 along with slack variables. The confusion matrix shown in Figure 10 shows a perfect accuracy rate with confusion observed only in the activities running and skateboarding as the model displayed false classification between these two action categories.

The recognition rate for the reference methods [113–116] is specified in Table 5.

Mironică et al. [113] proposed an approach to combine the frame features to model a global descriptor. The recognition accuracy of this method is affected when all the features are aggregated within a single descriptor and the BoW representation. Le et al. [114] proposed an unsupervised feature learning technique to learn the features directly from the video. They also explore an extended version of the ISA algorithm for learning the spatial-temporal features from the unlabeled data. The classification was performed using a multiclass SVM where the labels are predicted for all clips except the flipped versions resulting in a drop in accuracy.

**Figure 11:**
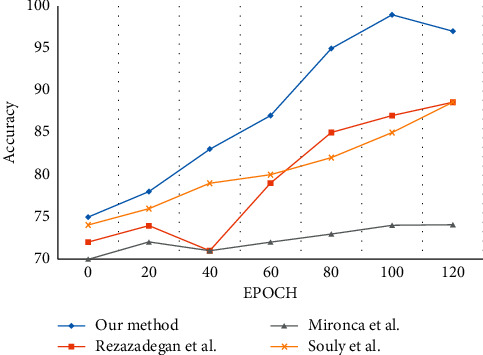
Evaluation of the classification accuracy for UCF sports action dataset.

An action region proposal method was provided by Rezazadegan et al. [115] using optical flows. Action detection and recognition were performed using CNN based on pose appearance and motion. Souly et al. [116] proposed an unsupervised method for detection using visual saliency [117] in videos. The video frames are divided into nonoverlapping cuboids and segmented using hierarchical segmentation to obtain the supervoxels from the cuboids. The features are decomposed into sparse matrices using PCA. When compared with the reference methods, the proposed method shows a better accuracy rate, as shown in Figure 11.

### 5.4. KTH Action Dataset

**Figure 12:**
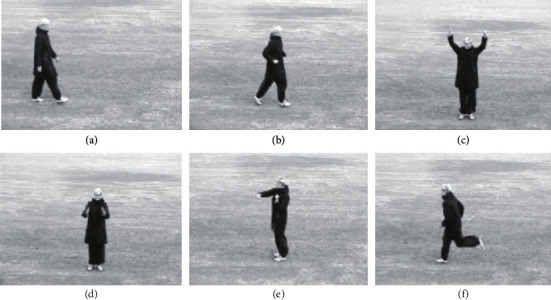
Actions considered for the proposed approach in the KTH dataset: (a) walking; (b) jogging; (c) waving; (d) clapping; (e) boxing; (f) running.

The KTH action dataset [102] is collated by the KTH Royal Institute of Technology. It is a video database that is comprised of human actions captured in various scenarios. It consists of six actions that include walking, boxing, running, waving, jogging, and clapping. The dataset is comprised of 600 video files that are a combination of 25 individuals, 6 actions, and 4 different types of scenarios, as shown in Figure 12.

**Figure 13:**
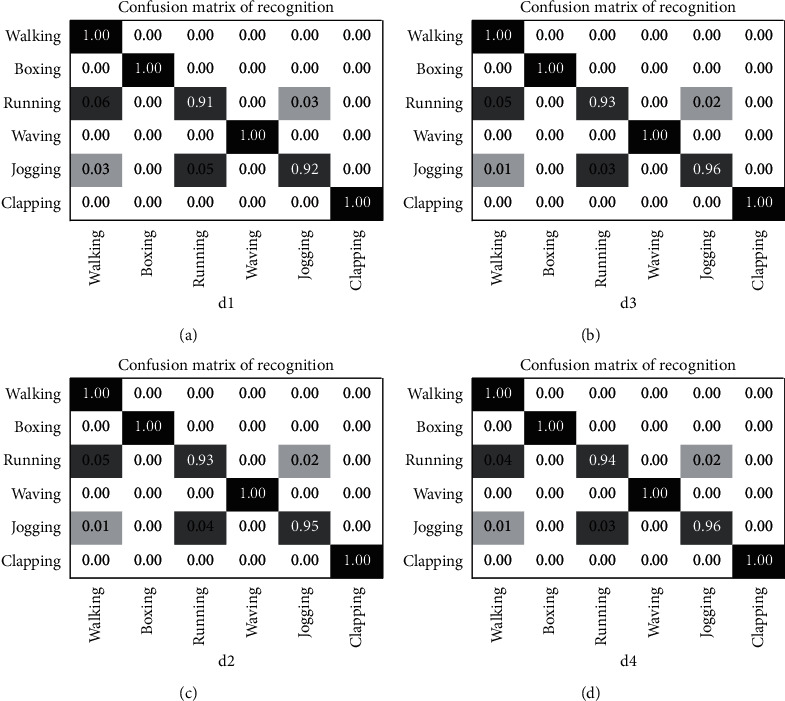
Confusion matrix for the KTH dataset with labels (a) d1, (b) d2, (c) d3, and (d) d4.

The experimental analysis is carried out using the reference methods [118–122]. Only one-third of the video samples are considered for experimentation. The 200 video samples are divided into 140 samples for the training set and 60 samples for the testing set. The confusion matrix for the dataset is shown in Figure 13. It can be observed that the classification rate was affected by the action category running, as it was detected as walking. The action category jogging was classified as running.

During experimentation, the parameter is fixed as *N* = 300 with four scenarios labeled as “d1,” “d2,” “d3,” and “d4.” The penalty coefficient is set as *N* = 10, and the slack variables are obtained by adaptive data matching. The average recognition rate for the dataset is shown in Table 6.

**Figure 14:**
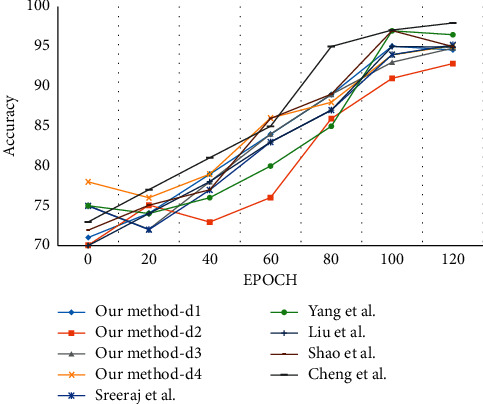
Evaluation of the classification accuracy for KTH dataset.

Sreeraj et al. [118] proposed a multiposture human detection system based on HOG and BO descriptors. This approach shows a slightly better accuracy rate as the system uses a fast-additive SVM classifier. This combined approach retains the HOG precision rate to improve the detection rate. Yang et al. [119] constructed a neighborhood by adding weights on the distance components. SONFs and MONFs are generated by concatenating multiple SONFs. The method also uses LGSR classifier for obtaining the multiscale-oriented features and achieves better classification. Ji et al. [120] proposed an improved interest point detection to extract the 3D SIFT descriptors from single and multiple frames by applying PCA. The quantification of combined features using SVM increases computational cost and causes a drop in accuracy rate. STLPC descriptor was proposed by Shao et al. [121] and learns the spatial-temporal features from the video sequence. A Laplacian pyramid is constructed by maxpooling to capture the structural and motion features efficiently. The proposed method shows a slight decrease in 0.11% and 1.4%. The classification accuracy for the KTH dataset is shown in Figure 14.

### 5.5. CASIA Action Dataset

**Figure 15:**
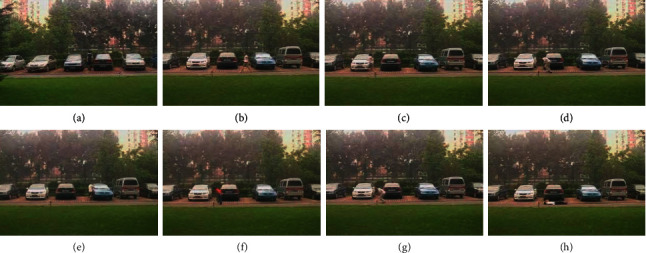
Action samples in the CASIA dataset: (a) wandering; (b) walking; (c) running; (d) crouching; (e) punching; (f) bending; (g) jumping; (h) falling.

The CASIA dataset [103] is comprised of 8 human actions such as running, walking, jumping, crouching, punching, wandering, bending, and falling. The video action sequences were captured using a static camera from various angles and views. There are 1446 video sequences performed by 24 different subjects, as shown in Figure 15. For the experimental analysis, 250 video sequences are analyzed. They are split into 190 samples for the training set and 60 samples for the testing set. The *N* parameter is set as 300 for every cell, while the penalty coefficient is set as *P* = 10 along with the respective slack variables. The reference framework [123] using the EM technique using an M-class SMV classifier and other classifiers is provided in Table 7.

**Figure 16:**
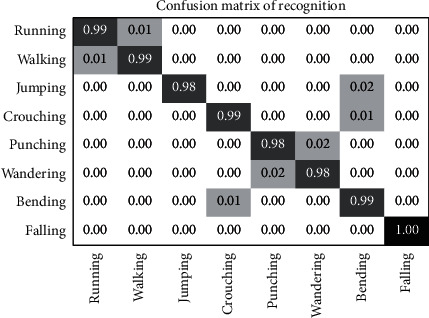
Confusion matrix for the CASIA dataset.

The confusion matrix in Figure 16 shows that the action category falling achieves a full accuracy rate. Similar action categories such as running, walking, crouching, and bending have a 99% accuracy rate. The categories of punching and wandering show the least accuracy rate of 98%.

Table 7 shows the average recognition rate for the CASIA dataset. Sharif et al. [123] proposed a hybrid strategy for human action classified by the integration of four major techniques. Initially, the objects in motion are uniformly segmented, and the features are extracted using LBP, HOG, and Haralick features. The feature selection is performed by the joint entropy-PCA method, and the classification is performed using multiclass SVM. The following classifiers multiclass SVM, DT, LDA, KNN, and EBT are used for experimental analysis. If high-resolution videos are used, there is a drop in efficiency due to computation overhead.

**Figure 17:**
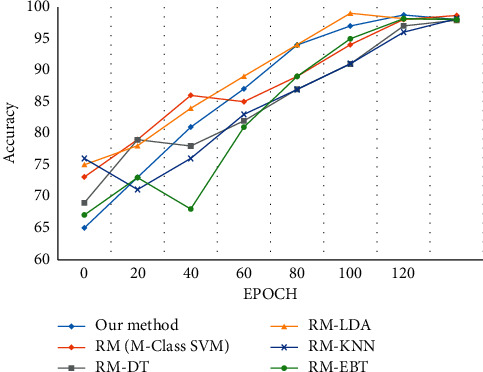
Evaluation of the classification accuracy for CASIA dataset.

Figure 17 shows that our proposed method has a better recognition rate when compared to the classifier used in the reference method.

### 5.6. i3DPost Multiview Dataset

**Figure 18:**
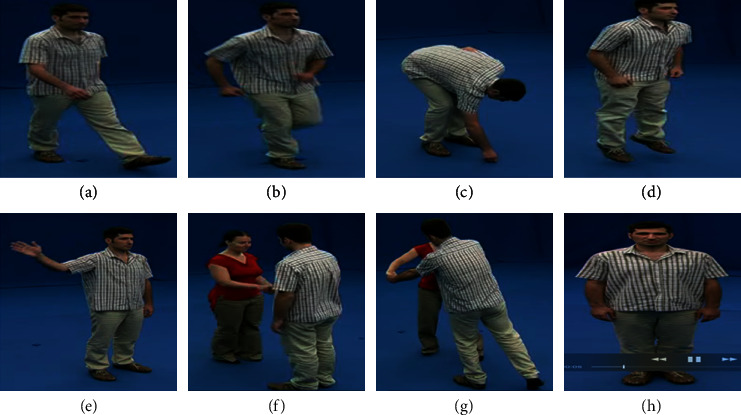
Actions considered in the i3DPost multiview dataset: (a) walking; (b) running; (c) bending; (d) jumping; (e) waving; (f) handshaking; (g) pulling; (h) face expressions.

The i3DPost dataset is a multiview/3D human action/interaction database [104] created by the University of Surrey and CERTH-ITI (Center of Research and Technology Hellas Informatics and Telematics Institute). The dataset consists of multiview videos and 3D posture model sequences. The videos were recorded using the convergent eight-camera setup for capturing high-definition images with twelve people performing twelve different types of human motions. The actions performed by the subjects include walking, running, bending, jumping, waving, handshaking, pulling, and facial expressions, as shown in Figure 18. The 104 video sequences are divided into 60 samples for the training set and 44 samples for the testing set. This is because the action in this dataset is much more complex than the UCF sports action dataset. The *N* parameter is set as 150 for every cell, while the penalty coefficient is set as *P* = 10 along with the respective slack variables.

**Figure 19:**
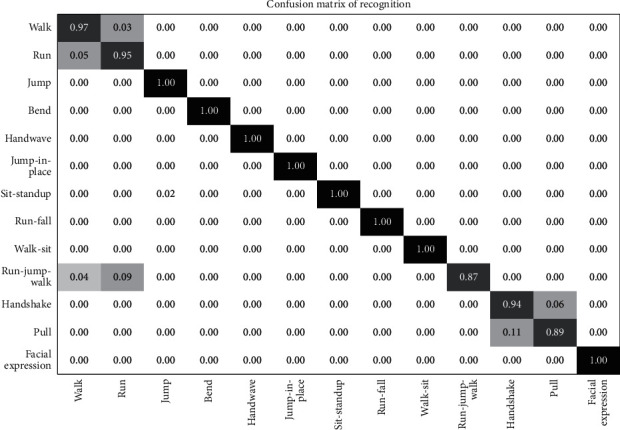
Confusion matrix i3DPost multiview dataset.

The confusion matrix obtained in Figure 19 shows that action categories jumping, bending, waving, stand-up, run-fall, and walk-sit have a full recognition rate. The actions running and walking have a misclassification rate in a few scenarios. Also, the actions handshaking and pulling are misclassified due to similar poses in some frames leading to a decrease in recognition rate.

In Table 8, Gkalelis et al. [124] and Iosifidis et al. [125] proposed an approach using binary masks obtained from multiview posture images for vectorization. This technique was used to extract the low-dimensional feature descriptors. DFT, FVQ, and LDA are applied for action recognition and classification. The authors tested their method with a limited testing set comprising only eight actions when compared to 13 actions used in our proposed approach.

**Figure 20:**
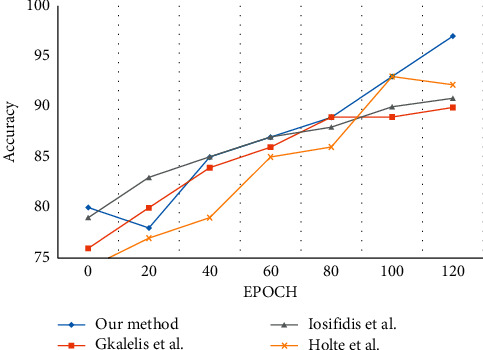
Evaluation of the classification accuracy for i3DPost multiview dataset.

Holte et al. [126] proposed a score-based fusion technique for extracting the spatial-temporal features. These feature vectors are efficient for high frame data capture with different densities and views. Based on the evaluation of the accuracy rate in Figure 20, the proposed method achieves significant performance when compared to other reference methods with 13 actions.

### 5.7. JHMDB Action Dataset

**Figure 21:**
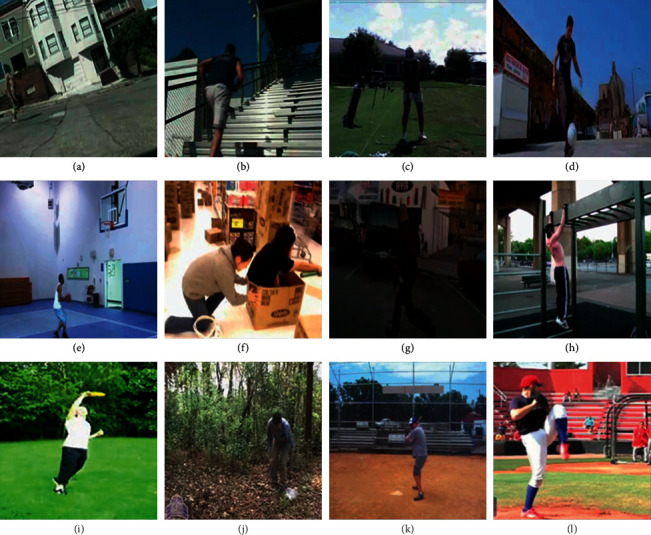
Actions considered for training in the JHMDB action dataset: (a) walking; (b) climbing; (c) golfing; (d) kicking; (e) jumping; (f) pushing; (g) running; (h) pull up; (i) catching; (j) picking up; (k) baseball playing; (l) throwing.

The joint-annotated human motion database [105] is categorized into 12 action types. The twelve actions shown in Figure 21 include walking, climbing, golfing, kicking, jumping, pushing, running, pull-up, catching, picking-up, baseball playing, and throwing.

The dataset comprises of three segmentation methods for the training and the testing set. For our experimentation, we are using only one segmentation method where only 316 videos are considered. They are further divided into 224 video segments for the training set and 92 video segments for the testing set. The *N* parameter is set as 350 for every cell, while the penalty coefficient is set as *P* = 10 along with the respective slack variables.

**Figure 22:**
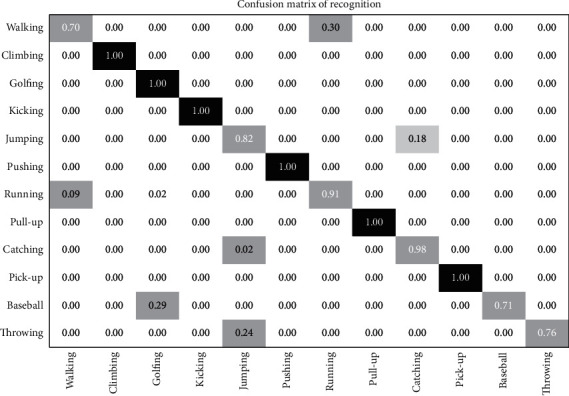
Confusion matrix for JHMDB action dataset.

The confusion matrix from Figure 22 shows that the action categories climbing, golfing, kicking, pushing, pull-up, and pick-up have a 100 percent recognition rate. The action categories such as jumping, running, and catching showed recognition rates ranging from 91 to 98 percent. The action categories that showed the least performance were walking that was misclassified with running. The action jumping was misclassified as catching and vice versa, while the action baseball playing was misclassified as golfing.

From Table 9, Jhuang et al. [105] performed a systematic performance evaluation using the annotated dataset. The baseline model was evaluated by categorizing the poses in the sample into three categories: low-, middle-, and high-level features. The dataset is annotated using a 2D puppet model, and the optical flow or the puppet flow is computed. The low- and mid-level poses are evaluated using the dense trajectory technique, while the high-level poses are evaluated using NTraj. Yu et al. [127] proposed a multimodal three-stream network for action recognition. PoseConvNET is used for detecting the 2D poses using the 2D CMU pose estimator, and the interpolation method is introduced for joint completion. The analysis performed on the individual cues showed a less recognition rate when compared with the proposed method.

**Figure 23:**
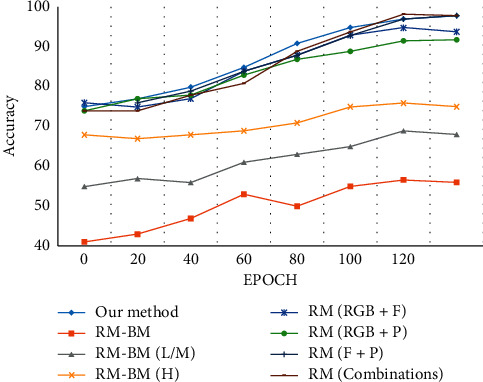
Evaluation of the classification accuracy for JHMDB action dataset.

However, when all the cues are combined, the reference method proposed by You et al. shows better recognition by 1.34 percent when compared to our proposed method. The evaluation of the accuracy rates for the model is shown in Figure 23.

### 5.8. UCF101 Action Dataset

**Figure 24:**
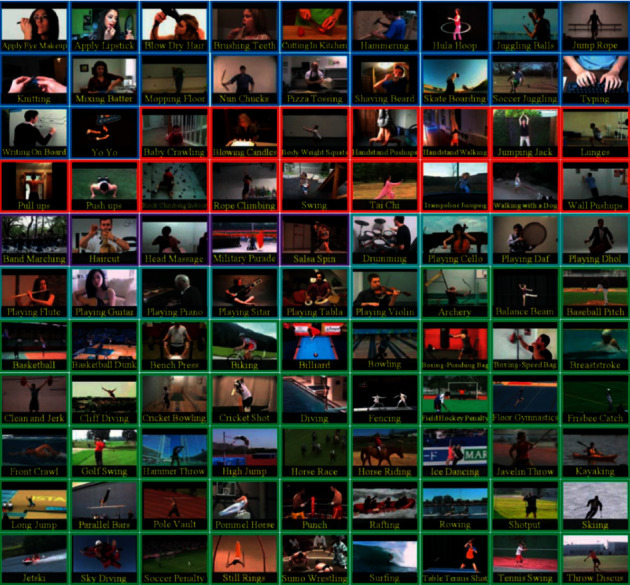
Action samples in the UCF101 action dataset.

The UCF101 [106] is a collection of human action dataset [128] and is an extended version of the UCF50 dataset. It is comprised of 101 human behaviors, and they are categorized into 25 groups, as shown in Figure 24. Every group is comprised of 13320 behavioral segment videos. The training and testing sets are divided into three categories. The average recognition rate from the three sets is analyzed from the dataset. The *N* parameter is set as 400 for every cell, while the penalty coefficient is set as *P* = 10, whereas other parameters are provided by pattern matching the image data to the processed image data.

The effectiveness of the algorithm is measured using the following reference algorithms [9, 93, 111, 129, 130], as shown in Table 10.

Ryoo [111] proposed a dynamic and integral BoW model for action prediction. The human activities are predicted using 3D spatial-temporal local features along with the interest points. The features values are clustered to form visual words using K-means and the Integral BoW used HOG descriptors. The method showed a drop in recognition rates during the early stages of detection. Cao et al. [129] proposed a probabilistic framework for action recognition. Sparse coding is applied to spatial-temporal features, and the likelihood is obtained using MSSC. The datasets were tested using SC and MSSC methods; the recognition rate was less satisfactory and required more training due to model complexity.

**Figure 25:**
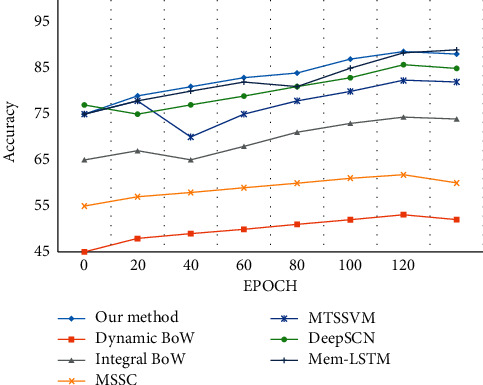
Evaluation of the classification accuracy for JHMDB action dataset.

Kong et al. [130] proposed the MTSSVM model for predicting the temporal dynamics of all the observed features. This approach showed an improvement in the recognition rate when compared to other reference methods. The drop in recognition rate is because the model requires prior knowledge of the temporal action that can be achieved only via prolonged training. A mem-LSTM model was proposed by You et al. [9] for recording the hard samples. The model used CNN and LSTM on the partially observed videos. The model has an improved recognition rate as it does not require prior knowledge of the features, and the global memory is sufficient for prediction. From Figure 25, it can be observed that the proposed method outperforms all the other reference methods.

### 5.9. HMDB51 Action Dataset

**Figure 26:**
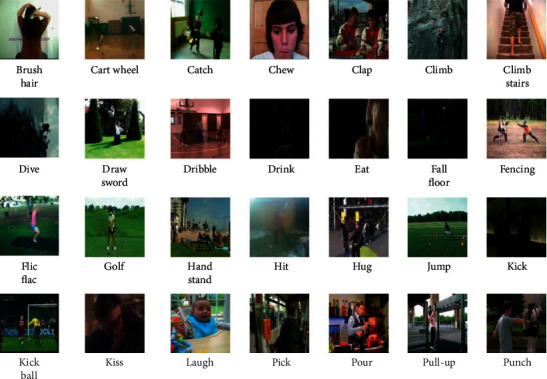
Action samples in the HMDB51 action dataset.

The HMDB51 action dataset [107] is comprised of 51 behavior categories that contain 100 videos each and 6676 action sequences, as shown in Figure 26. The data are divided into three training and testing sequences for action recognition, 60 training videos, and 30 test videos. From Table 11, the proposed method is evaluated with other techniques. The *N* parameter is set as 150 for every cell, while the penalty coefficient is set as *P* = 10.

Jiang et al. [131] proposed a fuss-free method for modeling motion relationships by adopting the global and locale reference points. The code words are derived from the local feature patches and tested. Jain et al. [48] proposed a technique for decomposing the visual motion into dominant motions to compute the features and their respective trajectories. A DCS descriptor along with the VLAD coding technique is used for action recognition.

Heng et al. [132] introduced a technique for matching the feature points between the frames using the SURF descriptor and optical flow. These matched features are graphed with RANSAC for human action recognition. Zhang et al. [133] proposed a deep two-stream architecture for action recognition using video datasets. The knowledge is transferred from optical CNN to motion vector CNN to reduce computation overhead and to boost the performance of the model.

**Figure 27:**
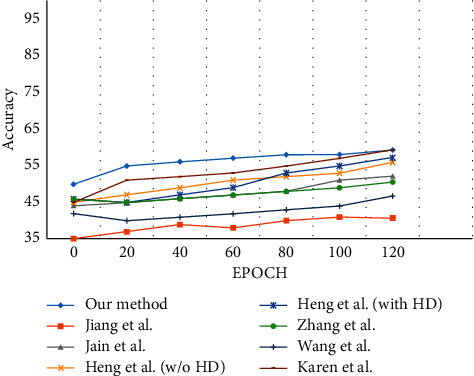
Evaluation of the classification accuracy for HMDB51 action dataset.

Karen et al. [135] proposed a two-stream ConvNet architecture to combine spatial-temporal features. The model is trained on dense multiframe optical flow to achieve enhanced performance.  Figure 27 shows that the proposed method surpasses all the techniques considered for evaluation.

### 5.10. Influence of the **N** Parameter, Model Accuracy, and Loss Function

Restricted Boltzmann machine (RBM) is a stochastic autoencoder that functions as both encoder and decoder. It is used for weight initialization in a neural network before training using stochastic gradient descent (SDG) for backpropagation. During training, multiple RBMs are stacked on top of each other to form a neural network. The RBM layer in the neural network inherits the functionality of the network. Thus, it can function as both an autoencoder or as a part of the neural network. As mentioned earlier, the RBM-NN comprises a two-layer neural network that is fully connected to other layers. The visible layer functions as the input layer, and the hidden layer corresponds to the features of the input neurons. During training, the RBMs adjust their weights automatically. The weight fed to one output neuron corresponds to one feature of the input. For instance, each weight originates from an input pixel, and the value determines the strength of the connection towards the activation function. The parameters generated by RBM are dynamic, and minor changes can cause huge differences in network behavior and performance. Every neuron is assigned to an activation function, and the node output is either set as 1 (on) or 0 (off).

**Figure 28:**
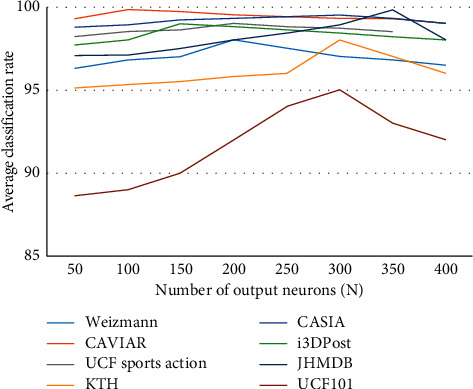
Influence of *N* parameter on the classification rate.

From Figure 28, we can observe that the classification accuracy of the model is influenced by the number of neurons provided to the RBM. The classification rate reaches the highest when it satisfies the *N* parameter and gradually decreases after crossing the threshold layer. The influence of the parameter for the all the datasets shows similar results.

**Figure 29:**
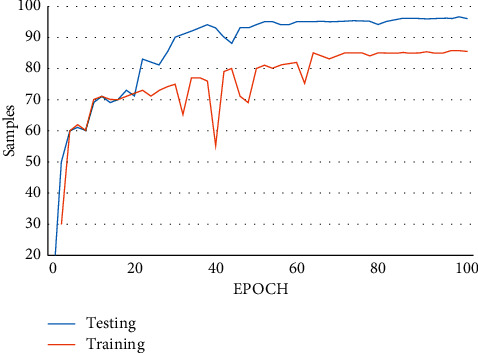
Model performance for the training and testing sets.

Deep learning neural networks are trained using the SDG optimization algorithm. As a part of the optimization problem, it is essential to evaluate the error rate for the current state of the model continuously. The error function used for our proposed method is a logistic regression loss function that estimates the loss of the models for weight updation. The loss function for our model is evaluated by generating a regression problem with a set of input variables, noise, and other properties. For evaluation, 100 input features are defined as input to the model. A total of 1000 samples will be randomly generated, and the pseudorandom number generator is fixed to 1 to ensure that the same number of samples is considered every time the model is evaluated. Each input and the output variable follows Gaussian distribution for data standardization. The model has the learning rate set to 0.1 with learning momentum set to 0.9. The model is trained for 100 epochs, and the testing set is evaluated at the end of every epoch to compute the loss function for the model.  Figure 29 shows the performance of the model for the training and testing sets. Since the input and target variable for the model follow Gaussian distribution, the average of the squared differences between the actual and predicted values are computed.

**Figure 30:**
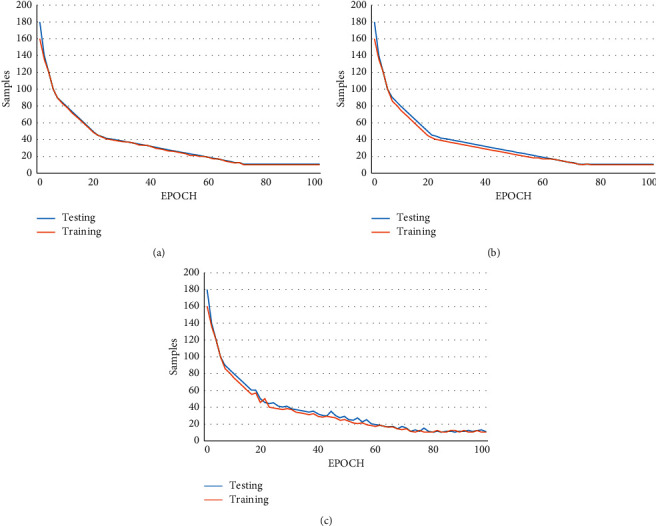
(a) Mean squared error (MSE) loss over training epochs; (b) mean squared logarithmic error (MSLE) loss over training epochs; (c) mean absolute error (MAE) loss over training epochs.

If the difference is large, a strict penalty is enforced on the model for making a misclassification. From Figure 30(a), we observe that model was capable of learning the problem by achieving near-zero error for MSE loss. The model converges reasonably for the training and the testing set with a good performance rate.

In case, if the target value consists of widespread values or the difference is large, punishing the model by enforcing a large penalty may affect the performance of the model. To avoid performance issues, the logarithm value for every predicted value is calculated, and then, the MSE is computed to obtain MSLE. MLSE reduces the penalty enforced on the model if a large spread of values is obtained. The same configuration is followed, and the model is tested for widespread values using MSE and MLSE. From Figure 30(b), it can be observed that the MSE loss is significantly higher for the training and testing sets. This indicates that the model may be showing signs of overfitting as there is a significant drop in the beginning and the model starts to recover gradually. Moreover, convergence between the training and the testing set occurs at a later stage.

For cases with large or small values when compared to the mean value, the model might run into outliers. The mean absolute error loss is considered to be suitable for handling outliers. It is used for calculating the absolute difference between the target and the predicted values. In Figure 30(c), the training and the testing set do not converge, and numerous spikes in values are observed, making it not a good fit in the case of outliers.

**Figure 31:**
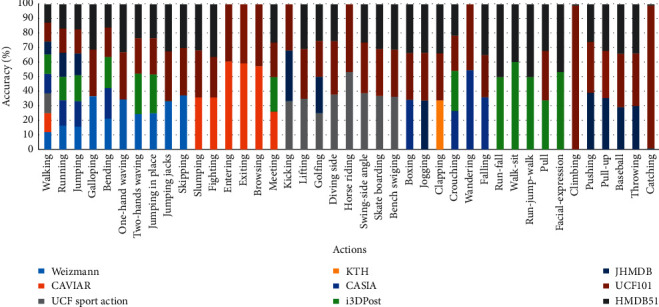
Performance evaluation in terms of accuracy of human action detection.

Figure 31 shows the overall performance evaluation of all the datasets that have been considered for human action recognition. The respective actions and the corresponding classification accuracy are provided for 41 action categories. For the training and testing, the individual actions such as walking, running, jumping bending, waving, jumping jacks, and skipping display better top-1 accuracy rates as the classification matches the target. However, combined actions such as run-fall, walk-sit, and run-jump-walk also show a better classification rate when compared to individual instances. The classification accuracy for standalone actions such as catching, entering, exiting, diving side, horse riding, skate boarding, facial expressions, and wandering was also classified accurately due to the probability of top-5 accuracy as the model considers the top five probabilities that match the target label.

The restricted Boltzmann machine is composed of binary visible units and binary hidden units. The parameters for the RBM are estimated using stochastic maximum likelihood (SML). The time complexity of the RBM network is estimated to be *O*(*n*), where *n* is considered to be the input features or the number of components. The parameters estimated using SML are the number of components, the learning rate for weight updation, batch size, number of iterations, verbose level, and random state. The random state determines the random number generation for sampling the visible and hidden layers and initializing the components required for sampling the layers during fitting. It also ensures that the data remain uncorrupted, and the scoring sample must obtain accurate results across multiple functions. The attributes considered for training the RBM are the biases of the hidden and visible units; the weight matrix and the hidden activation obtained from the model distribution are computed from the batch size and components.

Table 12 shows the computational complexity with respect to time for the various datasets. The table displays the dataset considered, number of videos, number of classes, pixel resolution, frames per second, the input sample considered for training the model, testing sample, training sample, testing and training accuracy, training time, and average epochs. From Table 12, it can be inferred that the training time increases when the video sample and the pixel resolution increase. The input samples are divided into mini batches and tested with various iterations. The training time after each iteration is recorded, and the time after individual iterations is averaged to obtain the training time of the dataset. The training time for JHMDB and UCF101 datasets is high as the input size and the pixel resolution are high. However, the training times of the datasets can be decreased, and better computation complexity can be achieved with better computational resources.

## 6. Conclusion

In this paper, a parameter adaptive initialization method that uses a neural network is proposed. The parameter initialization method is modeled based on Maxout activation function using RBM-NN. The spatial and temporal features are learned from various human action datasets. From the experimental analysis, the model learns the spatial-temporal features from the shape feature sequences. An RBM-based neural network model is designed with two layers, and an SVM classifier recognizes multiclass human actions. The proposed method is tested on various benchmark datasets and compared with existing state-of-the-art techniques. The experimental results showed that the proposed method accurately identifies various human actions. The recognition rate was found to be significantly better than other state-of-the-art specific and multiclass human action recognition techniques.

## Figures and Tables

**Table 1:** Comparison of test error rates of the initialization method.

Dataset	Test error rates	
	Xavier parameter initialization method (%)	Proposed parameter initialization method (%)
MS-COCO	10.25 ± 0.02	8.15 ± 0.09
CIFAR-100	19.38 ± 0.19	17.51 ± 0.22
ImageNet	23.19 ± 0.12	21.25 ± 0.17

**Table 2:** Action video datasets used in our proposed work.

Dataset	Year	Videos	Classes	Modality	Environment type
Weizmann [99]	2005	90	10	RGB	Controlled
CAVIAR [100]	2005	390	13	RGB	Controlled
UCF sports action [101]	2009	1,100	11	RGB	Uncontrolled
KTH [102]	2004	599	6	RGB	Controlled
CASIA [103]	2007	1446	8	RGB	Controlled
i3DPost [104]	2009	104	13	RGB	Controlled
JHMDB [105]	2011	316	12	RGB	Uncontrolled
UCF101 [106]	2012	13,320	101	RGB	Uncontrolled
HMDB51 [107]	2011	7000	51	RGB	Uncontrolled

**Table 3:** Average recognition rate for Weizmann dataset.

Method type	Average recognition rate (%)
Reference method using TD [110]	94.44
Reference method using HOG [110]	97.77
Reference method using HOF [110]	96.66
Reference method using MBH [110]	95.55
Reference method using combined methods [111]	96.66
Proposed method	96.3

**Table 4:** Average recognition rate for CAVIAR dataset.

Method type	Average recognition rate(%)
Reference with MFS detector [112]	81.82
Reference with OpenCV detector 20 [112]	79.84
Reference with OpenCV detector 25 [112]	73.61
Proposed method d1	99.14
Proposed method d2	99.28

**Table 5:** Average recognition rate for UCF sports action dataset.

Method type	Average recognition rate (%)
Rezazadegan et al. [115]	93.3
Mironică et al. [113]	74.1
Souly et al. [116]	88.6
Le et al. [114]	86.8
Proposed method	98.2

**Table 6:** Average recognition rate for KTH dataset.

Method type	Average recognition rate (%)
Sreeraj et al. [118]	95.21
Yang et al. [119]	96.50
Ji et al. [120]	94.92
Shao et al. [121]	95
Proposed method d1	94.5
Proposed method d2	92.9
Proposed method d3	94.8
Proposed method d4	95.1

**Table 7:** Average recognition rate for CASIA dataset.

Method type	Average recognition rate (%)
Reference framework with M-class SVM [123]	98.70
Reference framework with DT [123]	97.90
Reference framework with LDA [123]	98.20
Reference framework with KNN [123]	98.10
Reference framework with EBT [123]	98.10
Proposed method	98.75

**Table 8:** Average recognition rate for i3DPost multiview dataset.

Method type	Average recognition rate (%)
Gkalelis at al. [124] (with 8 actions)	90.00
Iosifidis et al. [125] (with 8 actions)	90.88
Holte et al. [126] (with 8 actions)	92.19
Proposed method (with 13 actions)	97.07

**Table 9:** Average recognition rate for JHMDB action dataset.

Method type	Average recognition rate (%)
Reference baseline model [105]	56.6
Reference baseline with low/mid-level pose [105]	69.00
Reference baseline with high-level pose [105]	76.00
Reference method with RGB + flow [127]	95.04
Reference method with RGB + pose [127]	91.67
Reference method with flow + pose [127]	97.10
Reference method with all combinations [127]	98.41
Proposed method	97.07

**Table 10:** Average recognition rate for UCF101 action dataset.

Method type	Average recognition rate (%)
Dynamic BoW [111]	53.16
Integral BoW [111]	74.39
MSSC [129]	61.79
MTSSVM [130]	82.39
DeepSCN [93]	85.75
Mem-LSTM [9]	88.37
Proposed method	88.64

**Table 11:** Average recognition rate for the HMDB51 dataset.

Method type	Average recognition rate (%)
Jiang et al. [131]	40.7
Jain et al. [48]	52.1
Heng et al. [132] without HD	55.9
Heng et al. [132] with HD	57.2
Zhang et al. [133]	50.6
Wang et al. [134]	46.7
Karen et al. [135]	59.4
Proposed method	59.21

**Table 12:** Computational complexity with respect to time for the various datasets.

Dataset	Classes	Resolution	Frames per second (fps)	Input video sample	Training set sample	Testing set sample	Testing accuracy (%)	Training accuracy (%)	Training time	Average epochs
Weizmann	10	180 × 144	25	91	60	31	94.1	96.3	53.05	60
CAVIAR	13	384 × 288	25	29	20	9	97.4	99.1	47.08	40
UCF SA	11	720 × 480	10	150	102	4	94.6	98.2	74.87	39
KTH	6	160 × 120	25	200	140	60	94.52	98.33	102.12	59
CASIA	8	320 × 240	25	250	190	60	95.7	98.75	146.12	75
i3DPost	13	1920 × 1080	15	104	60	44	93.19	97.09	51.91	40
JHMDB	12	320 × 240	25	316	224	92	88.71	90.83	203.72	45
UCF101	101	320 × 240	25	600	400	200	84.21	88.64	283.87	80
HMDB51	51	320 × 240	30	90	60	30	82.11	87.12	54.12	40

## Data Availability

The image datasets used to support the findings of this study are included in the article.

## List of Abbreviations

RBM: Restricted Boltzmann machines
RBM-NN: Restricted Boltzmann machines-neural network
MAF: Maxout activation function
SDG: Stochastic gradient descent
MSE: Mean squared error
MSLE: Mean squared logarithmic error
MAE: Mean absolute error.
